# Clinical and ultrasound features of fibrous pseudotumor of tunica vaginalis of the testis: eight cases and literature review

**DOI:** 10.3389/fonc.2024.1485723

**Published:** 2024-12-24

**Authors:** Jiali Yang, Hongyan Chen, Hualin Yan, Yue Zhang, Juxian Liu

**Affiliations:** Department of Ultrasound, West China Hospital of Sichuan University, Chengdu, Sichuan, China

**Keywords:** fibrous pseudotumor, testis, epididymis, tunica vaginalis, scrotum, ultrasound, clinical

## Abstract

**Objective:**

To investigate the clinical and ultrasound features of fibrous pseudotumor of tunica vaginalis of the testis

**Methods:**

The clinical and ultrasound features of fibrous pseudotumor of the tunica vaginalis diagnosed by pathology in West China Hospital of Sichuan University from 2006 to 2023 were retrospectively analyzed.

**Results:**

The study included 8 patients diagnosed with fibrous pseudotumor of the tunica vaginalis. The average age was 51.8 ± 17 years(range:25 to 80 years). Painless nodules or masses were present in 87.5% of cases, while 12.5% presented with painless scrotal enlargement. Ultrasound findings were as follows: Lesions were nodular in 7 cases and diffuse in 1 case (left side 50%, right side 50%). 75% involved the tunica vaginalis wall; 25% involved the epididymis with concurrent epididymitis. The tunica vaginalis wall on the affected side was significantly thicker than the contralateral side (4.58 ± 2.19 mm vs. 2.59 ± 0.48 mm, *P*=0.012). Hydrocele was present in 62.5% of the affected cases, poor sound transmission was noted in 62.5%, and septation was observed in 12.5%. 62.5% of cases exhibited multiple small solid nodules, with a maximum diameter ranging from 8∼19 mm. Nodules were well-circumscribed, regularly shaped and isoechoic or slightly hyperechoic. In 37.5% of cases, adjacent nodules were fused and 37.5% exhibited posterior attenuation. Punctate calcifications were present in 25% of cases. There is usually less blood flow in the lesion.

**Conclusion:**

This study demonstrates that fibrous pseudotumor of the tunica vaginalis is a rare scrotal disease affecting primarily middle-aged and elderly men. It typically presents unilaterally and carries a favorable prognosis following surgical treatment. Ultrasound commonly reveals multiple slightly hyperechoic or isoechoic solid nodules with ipsilateral thickening of the tunica vaginalis wall. In some cases, nodules may involve the epididymis, with associated epididymitis, fusion of adjacent nodules, occasional calcification, and posterior echo attenuation. Most lesions exhibit poor blood flow, and hydrocele is frequently present on the affected side. The distinct clinical and ultrasound features of the disease make non-radiative ultrasound imaging an effective tool for rapid detection, differential diagnosis, and guidance for appropriate clinical treatment.

## 1 Introduction

Fibrous pseudotumor of the tunica vaginalis, first described by Balloch in 1904 (1), is a rare, benign fibroproliferative lesion that arises from paratesticular tissues. It most often oraginates from tunica vaginalis, occasionally from epididymis, and rarely from spermatic cord and tunica albuginea (2). It typically presents as long-standing, painless scrotal mass (3). The lesions appear as multiple gray and white nodules with thickened fibrous bands surrounding the testis (4). The specific cell type responsible for these lesions has yet to be determined. In 1973, Mostofi and Price used the term “fibrous pseudotumor,” which has been adopted by most standard pathology textbook and guidelines (2). In the absence of imaging or preoperative biopsy evidence or intraoperative frozen section, scrotal space-occupying lesions are easily misdiagnosed as rhabdomyosarcoma or other malignant tumors, which may lead to unnecessary radical or total resection. there are few reports on imaging diagnosis and analysis of this condition in the English literature. Only four cases involving (Magnetic resonance imaging, MRI) have been reported (5–7), and its diagnostic characteristics remain unclear. Additionally, only a dozen cases involving ultrasonography have been documented (8–10), leaving the overall diagnostic information on this condition insufficient. There is also no literature available on the (Computed tomography, CT) diagnosis of this disease.

High-frequency color Doppler flow imaging (7, 9) is the preferred method for observing, diagnosing, and differentiating scrotal lesions due to its advantages, including lack of radiation, convenience, quick examination, high resolution, and real-time dynamic observation of the lesion’s location, size, echo, blood flow, and its relationship with surrounding structures and tissues. This study analyzes and summarizes the clinical and ultrasonic features of eight cases that were pathologically confirmed to be fibrous pseudotumors of the tunica vaginalis after surgery. The aim is to enhance the diagnostic and differential diagnostic capabilities of ultrasound for this disease and to provide important contributions to preoperative imaging diagnosis. It is necessary to avoid the misdiagnosis of malignant tumors before surgery before operation, which may lead to excessive treatment such as radical resection of the testis and epididymis.

## 2 Materials and methods

### 2.1 General information and ultrasound imaging data

This retrospective study was approved by the institutional review board of West China Hospital, Sichuan University (No.2440). As it was a retrospective study, the requirement for informed consent was waived. Clinical information, ultrasound images, and reports from eight male patients diagnosed with fibrous pseudotumor of the tunica vaginalis through postoperative pathology were retrospectively collected. These patients underwent ultrasound examination and surgery at West China Hospital of Sichuan University between 2006 and 2023. Data were retrieved from the hospital’s HIS system and ultrasound image database. Two senior residents collected and recorded the relevant data, with final confirmation by a senior specialist in cases of disagreement. The cases were numbered in chronological order based on the time of diagnosis.

### 2.2 Instruments and Methods

Ultrasound examinations were performed using Philips HD11, Philips HDI 5000, and Philips iU22 machines with 5-12 MHz linear array probes. Color Doppler velocity scale was adjusted 3 cm/s and optimized the color gain to eliminate artifacts. Ultrasound was used to assess the shape, size, echotexture, scrotal wall, spermatic cord, and blood flow of the bilateral testis and epididymis. Specific attention was given to the anatomical location of the lesions, the number of lesions, their relationship with the testis and epididymis, their boundaries, shape, size, internal echogenicity (including posterior acoustic attenuation, calcification, and blood flow), and the thickness of the tunica vaginalis and presence of hydrocele. The grading of blood flow was determined using Adler’s criteria: Grade 0: No blood flow signal detected within the lesion. Grade I: Minimal blood flow, with 1 to 2 punctate or thin rod-like vessels; rod-like blood flow does not exceed half the lesion’s diameter. Grade II: Moderate blood flow, with 3 to 4 punctate vessels or one long vessel penetrating the lesion. Grade III: Extensive blood flow, characterized by ≥5 punctate vessels or the presence of two long vessels (11). In this study, lesions with Adler blood flow grades 0 to II were categorized as having poor blood flow, while lesions with grade III were considered to exhibit abundant blood flow signals.

### 2.3 Statistical methods

SPSS version 25.0 and ImageJ statistical software were used for data analysis. Continuous data following a normal distribution were expressed as mean ± standard deviation. The sheath wall thickness was measured using ImageJ software, and the difference between the affected side and the contralateral side was evaluated using the Wilcoxon rank test. A *P*-value of less than 0.05 was considered statistically significant.

## 3 Results

A total of eight male patients with pathologically confirmed fibrous pseudotumor of the tunica vaginalis of the testis, complete medical records, and ultrasound data were collected for this study. The mean age of the patients was 51.8 ± 17 years (range: 25-80 years), and the mean disease duration ranged from over 2 months to over 10 years. Seven patients presented with painless testicular nodules without obvious causes, while one case presented with scrotal enlargement, although no definitive diagnosis was made preoperatively. Five patients underwent unilateral radical orchiectomy, and three underwent nodular and involved tunica vaginalis resection. During follow-up, no related complications or recurrence were observed.

**Figure 1:**
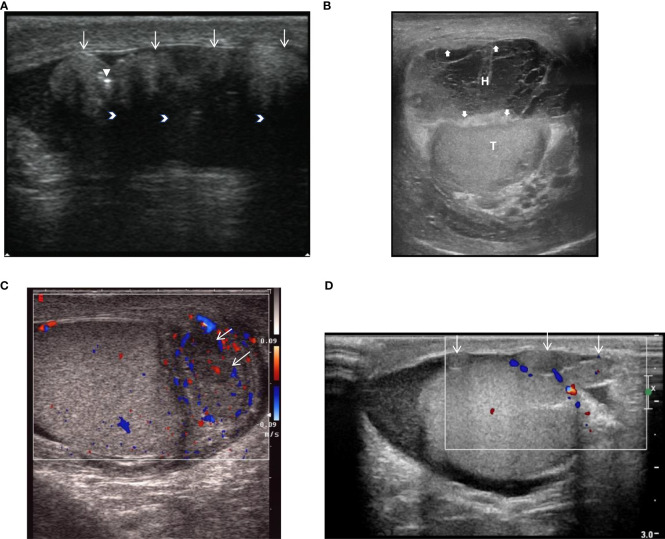
(Ultrasound) **(A)**. Male, 46 years old, Nodular fibrous pseudotumor, Ultrasound shows multiple slightly hyperechoic nodules with involvement of the tunica vaginalis (White slender point),nodules were fused with attenuation behind the nodules (white arrow), and a few punctate calcifications within the lesion (white triangle). **(B)**. Male, 80 years old, diffuse fibrous pseudotumor, ultrasound showing diffuse thickening of the vaginalis wall (white thick arrow), intrathecal thickening separates hydrops (H) and pushing testis (T). **(C)**. Male, 50 years old, nodular fibrous pseudotumor of tunica tunica testis in cauda epididymidis with epididymitis. Ultrasound showed that the cauda epididymidis was enlarged, with 2 hypoechoic nodules (white slender arrows) in size, and rich blood flow signals in the epididymis. **(D)**. Case8, male, 40 years old, nodular fibrous pseudotumor, ultrasound clearly showed multiple isoechoic nodules (white slender arrows) in the wall of the sheath without abundant blood flow signals.

The ultrasound findings of these eight cases of fibrous pseudotumor of the tunica vaginalis are summarized in **Table 1** and depicted in **Figure 1**. Seven cases presented with a nodular type, while one case exhibited a diffuse type (Case7). Six of the lesions were localized to the tunica vaginalis, with four cases also involving the epididymis. The ultrasound findings in five of these cases revealed multiple small, solid, oval nodules (**Table 2**). The maximum nodule diameter ranged from 8 mm to 19 mm, with an average size of 13 ± 4.3 mm. Most nodules were slightly hyperechoic or isoechoic, with some exhibiting fusion and posterior attenuation. Only two cases demonstrated punctate calcifications. In terms of blood flow, most nodules exhibited poor blood flow signals, except for two cases involving the epididymis, which displayed rich blood flow signals and epididymitis. Additionally, hydrocele was present in five cases, and the thickness of the affected tunica vaginalis (3.0-9.6 mm, average: 4.58 ± 2.19 mm) was significantly greater than that of the contralateral side (2.1-3.4 mm, average: 2.59 ± 0.48 mm), with a statistically significant difference (Wilcoxon signed-rank test, *P* = 0.012, < 0.05). The diffuse fibrous pseudotumor of the tunica vaginalis (**Figure 1B**) exhibited diffuse thickening of the lateral tunica vaginalis, with nodular thickening in some regions, reaching a maximum thickness of approximately 9.6 mm (contralateral thickness of 3.4 mm) accompanied by viscous effusion (the range was about 52×33×36mm) of the tunica vaginalis, and the left testis and caput epididymidis were pushed backward. No abnormalities were identified in the testes or scrotal walls in any of the cases.

**Table 1:** Ultrasound findings of fibrous pseudotumor of tunica vaginalis (n=8).

Case	Age	Pathoge-nic site	Involved anatomical levels	sheath Thickness/(offside)	hydrocele	Lesion	Adler grade
			Tunica adnata testis(+) epididymis(+)		+/- sonolucency good(-)/poor(+) septation(+)	Nodule	
1	25	Right	+ −	4.5/(2.7)	− − −	+	2
2	50	Left	− +	4.7/3.2	− − −	−	3
3	69	Left	− +	3.2/2.4	+ + −	−	3
4	48	Right	+ −	3.3/(2.2)	+ + −	+	0
5	46	Right	+ −	5.1/(2.5)	+ + −	+	1
6	57	Right	+ +	3.0/(2.1)	+ + −	+	0
7	80	Left	+ −	9.6/(3.4)	+ + +	−	1
8	40	Left	+ +	3.2/(2.2)	+ + −	+	1
*P* value				0.012*			

*The difference of sheath thickness between the affected side and the contralateral side was *P*= 0.012.

**Table 2:** Ultrasound findings of fibrous pseudotumor nodules of vaginalis testis (n=5).

Case	Nodule number	Maximum nodule size(mm^3^)	solid	Clear boundary	Morphological rule	internal echo	Fusion of adjacent nodules(+)	Posterior attenuation(+/-)	Calcification(+/-)
1	2	19×12×16	yes	yes	yes	hyper	−	−	−
4	>2	13×8×10	yes	yes	yes	equal	+	+	−
5	>2	10×8×9	yes	yes	yes	hyper	+	+	+
6	>2	15×13×14	yes	yes	yes	equal	+	+	+
8	>2	8×3×7	yes	yes	yes	equal	−	−	−

**Figure 2:**
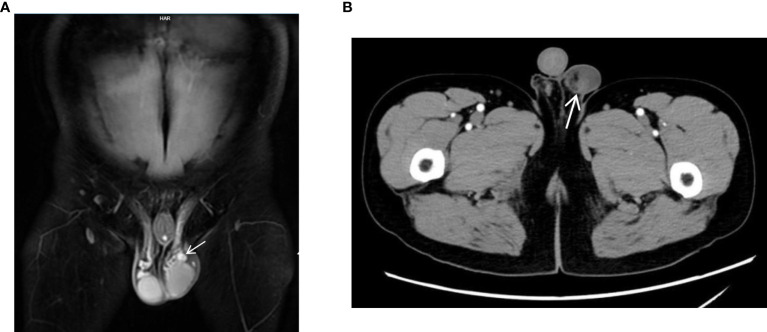
(MRI and CT). **(A)** Case8, coronal view of enhanced MRI shows a mixed signal nodule (white elongated arrow) in the left testicle sheath and epididymis, significantly enhanced. **(B)** Case8, enhanced CT cross-section shows a slightly dense nodule (white elongated arrow) above the left testicle which is seen as enhancement.

**Figure 3:**
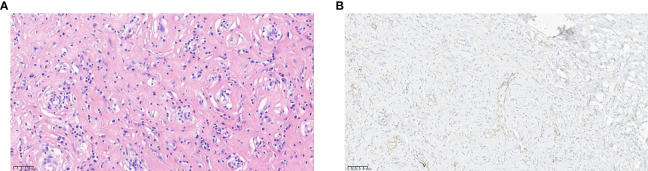
(Pathology) **(A)** Pathology of pseudomatosis fibrosus vaginalis. hematoxylin-eosin (HE) staining showed nodular proliferation of fibers/myofibroblasts in multiple focal areas of epididymis and sheath, extensive glass lesions with more inflammatory cell infiltration, and small vascular proliferation in some nodules (HP-×32). **(B)** Immunohistochemistry of fibrous pseudotumor. Visible proliferative fibers/myofibroblasts Desmin (+) (HP-×16).

Moreover, by comparing various imaging modalities (**Figures 1D**, **2A, B**) within the same case (Case8), we found that although enhanced MRI and CT were able to detect the lesions, ultrasound more clearly delineated the location, shape, size, boundary, internal echogenicity, and blood flow of the lesions. Pathological analysis of the surgical specimen from this case revealed nodular hyperplasia of fibrous/myofibroblastic cells in multiple foci within the epididymis and tunica vaginalis, extensive vitreous degeneration, significant inflammatory cell infiltration, and small vessel proliferation within some nodules. These findings were consistent with an inflammatory pseudotumor. Immunohistochemically, the proliferative fibrous/myofibroblasts were positive for desmin, further supporting the diagnosis of inflammatory pseudotumor (**Figure 3**).

Postoperative follow-up: The eight patients were followed for a period ranging from 3 months to 6 years. During this time, no related complications or signs of recurrence were observed.

## 4 Discussion

Testicular fibrous pseudotumor is a rare benign lesion of the scrotum, accounting for approximately 6% of benign lesions in the testicular adnexa, making it the third most common lesion in this region (8). It is characterized by the proliferation of fascicular fibroblasts/myofibroblasts and is often accompanied by chronic inflammatory cell infiltration, including plasma cells and mast cells. Multiple names have been applied for these leisions including fibroma, pseudofibromatosis periorchitis, inflammatory pseudotumor, and proliferative periorchitis. These names reflect its presentation, which is eithor that of multiple gray-white nodules within the tunica vaginalis (i.e., pseudotumor) or thickened fibrous bands surrounding the testis (i.e., periorchitis), which are indicative of specific inflammatory changes (4).

There have been few reports regarding the imaging diagnosis of this disease over the past two decades in the English literature. The disease generally affects middle-aged and elderly men, with an average disease duration of 4.6 years. In some cases, the disease can persist for an extended period without a definitive diagnosis. It typically presents unilaterally, with slow-growing, non-tender lesions that are often discovered incidentally during palpation (12). The clinical radioluminescence test is usually negative, and serum B-human chorionic gonadotropin and fetoprotein levels are within normal limits, which aids in distinguishing this condition from germ cell tumors (4).T Lesion sizes typically range from 0.5 to 8 cm, though, in rare cases, they can reach up to 25 cm (1). The condition is exceedingly rare in childhood, with only 8 cases reported in patients under 18 years old (2, 4, 6, 13).

In this study, seven patients presented with palpable nodules without any clear precipitating factors, while one patient had painless scrotal swelling. Most of the cases involved patients aged 40 years or older, with an average age of 51.8 ± 17 years, and only one patient was 25 years old. No pediatric cases were identified, aligning with findings in the literature. The disease duration ranged from just over two months to more than 10 years, with most cases lacking significant clinical symptoms. All cases were unilateral, with no notable difference in the occurrence between the left and right sides, consistent with previous reports. The size of the nodules in our study ranged from 8 mm to 19 mm, and all nodules were relatively small, with no large masses observed. Postoperative outcomes were favorable in all cases, with no recurrence noted during follow-up.

The etiology of testicular fibrous pseudotumor remains unclear, though some studies suggest associations with previous trauma, hydrocele, or infection (e.g., epididymitis) (9). There is consensus that hydrocele is the most frequent clinical manifestation of testicular fibrous pseudotumor (14). In our study, 75% of cases presented with a viscous effusion of the tunica vaginalis on the affected side, occasionally accompanied by septation, which is consistent with previous reports. Two cases involved the epididymis, both of which demonstrated abundant blood flow signals and were associated with epididymitis. Reports of fibrous pseudotumor exclusively affecting the epididymis are rare, but when the lesion does involve the epididymis, epididymitis may be present. Schistosoma haematobium infection has also been associated with fibrous pseudotumor, particularly in cases involving large or rapidly growing lesions (15). Other disease associated with fibrous pseudotumor include IgG-4-related immune disorders, such as retroperitoneal fibrosis, sclerosing cholangitis, sclerosing pancreatitis, and Riedel’s thyroiditis (16), but only a few cases have confirmed literature reports supporting such an association. No such associations were observed in our cohort.

Scrotal ultrasonography is the preferred imaging modality for detecting and evaluating scrotal lesions, offering precise anatomical details and the ability to distinguish between intratesticular and extratesticular abnormalities (7, 10). Some scholars classify fibrous pseudotumors of the tunica vaginalis into two types: nodular (characterized by discrete nodules) and diffuse (characterized by diffuse membrane thickening) (3, 17). Despite their differing appearances, both types share the same histopathological features. Some researchers posit that diffuse fibrous pseudotumor is a precursor to the nodular type, supported by the presence of small nodules within diffuse fibrous pseudotumors (2). Ultrasound of nodular fibrous pseudotumors typically reveals one or more solid masses near the testis, which may coalesce into larger formations. The nodules may appear well-demarcated or lobulated, or they may exhibit poorly defined borders (10). The echogenicity of the lesion varies depending on its fibrous and collagen content and the presence of calcifications, which can result in slightly hyperechoic or hypoechoic appearances. Dense fibers and collagen within the lesion can cause posterior attenuation, even in the absence of calcifications (4). In our study, five patients had solid nodules, which were either slightly echogenic or isoechoic. Three nodules exhibited posterior attenuation without calcification, and adjacent nodules had coalesced in three cases. In two cases, the lesions demonstrated small punctate calcifications. We hypothesize that the nodules contained dense fibers and abundant matrix collagen, which contributed to the slightly echogenic or isoechoic appearance, as well as attenuation despite the absence of calcifications. Therefore, the combination of slightly echogenic or isoechoic nodules, posterior attenuation, and fusion of adjacent nodules may serve as key diagnostic features of this condition on ultrasound. Previous studies have reported that color Doppler ultrasound often reveals no blood flow or only light to moderate flow in these lesions (10, 18). In our study, six cases demonstrated blood flow signals, while two cases showed abundant blood flow signals associated with epididymitis. The remaining lesions exhibited limited blood flow, consistent with findings in the literature.

Diffuse fibrous pseudotumor often presents with testicular enlargement and a hardened texture, making it difficult to distinguish from malignant testicular tumors. Palpation may reveal a hardened testis, possibly due to the diffuse proliferation of the tunica vaginalis (8, 17). In our study, all affected cases showed significantly thickened tunica vaginalis on the involved side compared to the contralateral normal side (*P* < 0.05%). Radical orchiectomy is often required in cases of diffuse fibrous pseudotumor (19, 20). One of our patients (Case7) presented with diffuse fibrous pseudotumor, characterized by left testicular enlargement and a hardened texture. Ultrasound revealed diffuse thickening of the testicular sheath on the affected side, with nodular thickening in some areas, viscous effusion separated by the testicular sheath, and displacement of the testis and epididymis. The patient ultimately underwent radical orchiectomy on the affected side. In future studies, ultrasound elastography could be employed to measure the stiffness in patients with testicular stiffness potentially providing valuable diagnostic information.

Based on our study and a review of the literature, we propose that fibrous pseudotumor of the tunica vaginalis exhibits the following clinical and ultrasound characteristics: it predominantly occurs in middle-aged and elderly men, typically presents unilaterally, and is usually treated surgically, with a favorable prognosis. Ultrasound commonly reveals multiple small nodules with thickening of the ipsilateral tunica vaginalis and hydrocele. Some nodules may involve the epididymis, and some lesions may exhibit echo attenuation. The majority of lesions are solid, slightly hyperechoic, or isoechoic, and some nodules may coalesce. Calcification is rare, and bilateral testicular and scrotal involvement is uncommon.

It is crucial to differentiate fibrous pseudotumor from other conditions, such as testicular appendage lesions, epididymitis, and rhabdomyosarcoma of the scrotal wall. (1) Testicular appendages, typically located at the upper pole of the testis, share a similar echogenicity with the testis and epididymis and are often difficult to detect via ultrasound. (2) Epididymitis commonly affects young, sexually active individuals aged 15 to 35 years and is often accompanied by pain. The epididymis is generally enlarged, with reduced and heterogeneous echogenicity. In severe cases, small abscesses with anechoic areas and abundant blood flow signals may be present, and the condition typically responds to anti-infective treatment. (3) Rhabdomyosarcoma of the scrotal wall is a rare malignant tumor of the male reproductive system that may present as a single or multiple lesions. The lesion often involves the testis and epididymis, with poorly defined margins, and may be accompanied by hydrocele. During examination, the lesion may appear as a large mass originating from the scrotal wall, with reduced echogenicity, absence of posterior attenuation, no calcification, and potential metastasis to abdominal and groin lymph nodes (21).

On MRI, fibrous pseudotumor of the testis typically exhibits a slightly hypointense signal on T1-weighted imaging (T1WI) and a markedly hypointense signal on T2-weighted imaging (T2WI) and fat-suppressed sequences (FS). T1WI lesions can show mild to moderate enhancement following contrast administration (7). On CT, the lesion may appear as a circular low-density mass with heterogeneous density, annular calcifications, and mild to moderate enhancement post-contrast. In Case 8, enhanced MRI and CT scans revealed nodules adjacent to the testis with enhancement within the nodules. However, neither approach allows to observe the relationship between the lesion and surrounding tissues in real time.In addition to being radiation-free and allowing repeated examination, ultrasound also has the advantages of real-time dynamic observation of lesion location, size, echogenicity, blood flow and its relationship with surrounding structures.Based on our study and literature review, this disease has distinct clinical manifestations and ultrasound characteristics, and ultrasound appears to offer greater diagnostic utility for this condition. It has also been pointed out that in cases where the lesion is so large that the relationship between the lesion and the surrounding tissue cannot be clearly defined by ultrasound, combined MRI scanning may provide some additional information to assist the diagnosis (5–7). In future studies, we hope that combined ultrasound and MRI diagnosis of the disease can provide more diagnostic information to assist the diagnosis.

Intraoperative consultation can guide the most appropriate treatment, though establishing a definitive diagnosis of fibrous pseudotumor during cryosectioning is challenging, often leading to a descriptive diagnosis (22). Most reported cases (2–4, 19) were diagnosed following radical orchiectomy. However, nodular fibrous pseudotumor of the tunica vaginalis can generally be resected, with the affected tunica vaginalis preserved. There is also some literature (13, 23, 24) suggesting that excisional biopsy or intraoperative frozen section evaluation for inguinal exploration may also avoid unnecessary radical orchiectomy.When preoperative ultrasound findings suggest the possibility of fibrous pseudotumor, a biopsy or intraoperative frozen section may be performed to confirm the diagnosis before surgery. If malignancy is ruled out, nodular fibrous pseudotumor can usually be excised locally, sparing the testis. Radical orchiectomy is typically unavoidable in cases involving diffuse fibrous pseudotumor (3, 18).

This study has several limitations. First, it covers a long time span, from 2006 to 2023, with a small number of cases. This is likely due to the rarity of fibrous pseudotumor of the tunica vaginalis, which results in a low incidence and consequently a small sample size. The largest lesion reported in the literature was 25 cm; however, most cases in this study involved multiple small solid nodules (ranging from 8 to 19 mm), which may not fully represent all the characteristics of this lesion. In future studies, it is recommended to increase the sample size in order to capture a broader range of imaging and clinical features of this disease. Additionally, only one case in this study was examined by MRI and CT before surgery. Previous studies have demonstrated that MRI is superior in the preoperative evaluation and postoperative follow-up of scrotal lesions (5–7). However, due to national conditions, the cost of MRI and CT is generally higher than that of ultrasound, which may have influenced the limited use of these modalities in this study. In future follow-up studies, obtaining more imaging data (including ultrasound, MRI, CT, etc.) would improve the clinical detection and diagnosis of this condition.

## 5 Conclusion

Fibrous pseudotumor of the tunica vaginalis is a rare scrotal condition typically affecting middle-aged and elderly men. It predominantly presents unilaterally and generally has a favorable prognosis following surgical intervention. Ultrasound imaging typically reveals multiple small nodules accompanied by thickening of the ipsilateral tunica vaginalis and effusion. In some cases, the nodules involve the epididymis, and certain lesions may exhibit echo attenuation. The majority of the lesions are solid or slightly hyperechoic, with occasional fusion of nodules and minimal calcification. Most lesions exhibit poor vascularization and do not extend to the tunica vaginalis wall or testis. The characteristic features observed on preoperative ultrasonography, combined with relevant clinical history, are valuable for the detection and diagnosis of fibrous pseudotumor. When malignancy has been definitively excluded, consideration of this condition may help to prevent unnecessary radical orchiectomy.

## Data Availability

The original contributions presented in the study are included in the article/supplementary material. Further inquiries can be directed to the corresponding author.
